# Development of new experimental platform ‘MARS’—Multiple Artificial-gravity Research System—to elucidate the impacts of micro/partial gravity on mice

**DOI:** 10.1038/s41598-017-10998-4

**Published:** 2017-09-07

**Authors:** Dai Shiba, Hiroyasu Mizuno, Akane Yumoto, Michihiko Shimomura, Hiroe Kobayashi, Hironobu Morita, Miki Shimbo, Michito Hamada, Takashi Kudo, Masahiro Shinohara, Hiroshi Asahara, Masaki Shirakawa, Satoru Takahashi

**Affiliations:** 1 Mouse Epigenetics Project, ISS/Kibo experiment, Japan Aerospace Exploration Agency (JAXA), Tsukuba, Japan; 2 JEM Utilization Center, Human Spaceflight Technology Directorate, JAXA, Tsukuba, Japan; 3 Department of Physiology, Gifu University Graduate School of Medicine, Gifu, Japan; 4 Department of Anatomy and Embryology, Faculty of Medicine, University of Tsukuba, Tsukuba, Japan; 5 Laboratory Animal Resource Center, Faculty of Medicine, University of Tsukuba, Tsukuba, Japan; 6 Transborder Medical Research Center, Faculty of Medicine, University of Tsukuba, Tsukuba, Japan; 7 Department of Systems BioMedicine, Graduate School of Medical and Dental Sciences, Tokyo Medical and Dental University, Tokyo, Japan; 8 Japan Science and Technology Agency (JST), Precursory Research for Embryonic Science and Technology (PRESTO), Tokyo, Japan

## Abstract

This Japan Aerospace Exploration Agency project focused on elucidating the impacts of partial gravity (partial *g*) and microgravity (μ*g*) on mice using newly developed mouse habitat cage units (HCU) that can be installed in the Centrifuge-equipped Biological Experiment Facility in the International Space Station. In the first mission, 12 C57BL/6 J male mice were housed under μ*g* or artificial earth-gravity (1 *g*). Mouse activity was monitored daily via downlinked videos; μ*g* mice floated inside the HCU, whereas artificial 1 *g* mice were on their feet on the floor. After 35 days of habitation, all mice were returned to the Earth and processed. Significant decreases were evident in femur bone density and the soleus/gastrocnemius muscle weights of μ*g* mice, whereas artificial 1 *g* mice maintained the same bone density and muscle weight as mice in the ground control experiment, in which housing conditions in the flight experiment were replicated. These data indicate that these changes were particularly because of gravity. They also present the first evidence that the addition of gravity can prevent decreases in bone density and muscle mass, and that the new platform ‘MARS’ may provide novel insights on the molecular-mechanisms regulating biological processes controlled by partial *g*/μ*g*.

## Introduction

Animals living on the Earth are subject to the effects of the Earth’s gravity (1 *g*). Hypergravity exceeding 1 *g* can be generated on Earth; however, the generation of gravity of <1 *g*, which is known as partial gravity (partial *g*) and microgravity (μ*g*), is not possible except in very short durations as generated by free drops or parabolic flight. To determine how gravity affects the development of animals and understand the process of adapting to partial *g*/μ*g* environments, animals would have to be raised on other planets or in the space.

Many µ*g* experiments using mice have been conducted in space aboard the Space Shuttles, such as ‘BION’, an unmanned satellite, and the International Space Station (ISS). The National Aeronautics and Space Administration (NASA), Russian Federation State Research Center Institute of Biomedical Problems RAS (IBMP) and the Italian Space Agency (ASI) have developed animal habitation cages for each space mission. The Animal Enclosure Module (AEM) developed by NASA was used for female mice in six Space Shuttle missions (STS-90, STS-108, STS-118, STS-131, STS-133 and STS-135) for approximately 2 weeks of habitation, and for female and male mice in four recent Rodent Research missions (RR-1 to RR-4) for up to 45 days of habitation^1–6^. Launched in 2013, the Russian Bion-M1 biosatellite accommodated male mice in groups for 30 days^7, 8^. The Italian Mice Drawer System (MDS) housed 6 male mice individually for 90 days on the ISS (STS-128/129)^9–11^. In the latter two experiments with male mice, more than half of the animals died because of hardware malfunctions or unpredictable reasons during habitation in space, suggesting that the control of habitat integrity is important for space mouse experiments. These space flight experiments have provided a broad range of results pertinent to biomedical applications, including neurology and muscle/bone physiology. Although results were informative, the comparable control 1 *g* experiments were conducted on the ground instead of in space. Various conditions differ between the ground and space environments, including cosmic radiation, microbial environment, and the lack of convection^12–14^. Moreover, flight mice experience vibration, shock and semi-steady acceleration during the launch and return phases inside the space vehicle. Thus, setting ground-housed mice as the 1 *g* control group may prevent a clear identification of the impact of μ*g* on mice. To elucidate the specific impacts of μ*g* on mice, we developed a novel experimental platform to generate artificial gravity (artificial *g*) in space.

**Figure 1:**
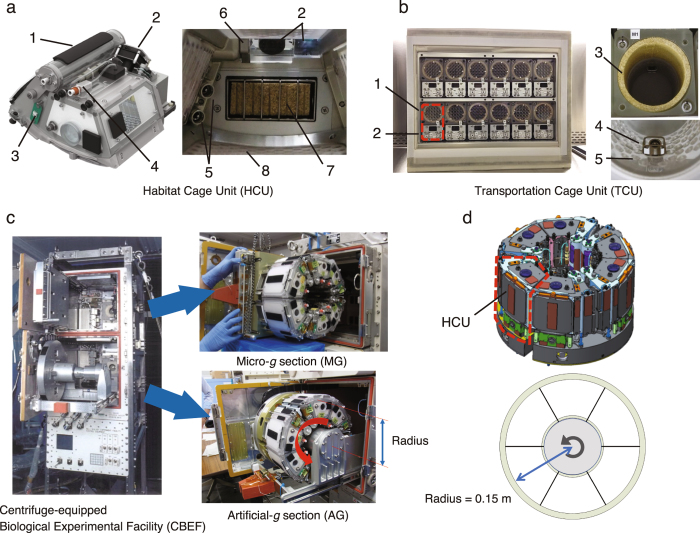
Newly developed mouse cages for the space experiment. (**a**) Habitat Cage Unit (HCU). The HCU accommodates one mouse per cage. 1: Water tank, 2: Camera/LED, 3: Temperature sensor, 4: Washer water inlet, 5: Water nozzles, 6: Wiper for camera, 7: Food cartridge, 8: Polycarbonate floor/walls. A 7-day supply of food and water is provided inside the HCU. (**b**) Transportation Cage Unit (TCU). The TCU contains 12 cylindrical cages in one unit. Each cylindrical cage accommodates one mouse. The TCU supports up to a 10-day supply of food and water during the launch and landing phases. 1: Housing area with food, 2: Water tank, 3: Food, 4: Water nozzle, 5: Polycarbonate floor/walls. (**c**) Centrifuge-equipped Biological Experiment Facility (CBEF). The CBEF has two compartments: the micro-*g* section and the artificial-*g* section with a centrifuge. (**d**) AG section. The centrifuge accommodates six HCU and the rotation radius where it contacts the floor is 0.15 m.

The Japanese Experimental Module (called ‘Kibo’) on the ISS began to be used for research in 2008, and numerous experiments were conducted using cultured cells, worms, aquatic organisms and plants, however none were performed using mice. Kibo experiments use the Centrifuge-equipped Biological Experiment Facility (CBEF), which provides both μ*g* and artificial *g* environments. As this compact onboard centrifuge is only 0.3 m in diameter, we consequently developed new mouse Habitat Cage Units (HCU) (Fig. 1a). We also developed the Transportation Cage Unit (TCU), in which mice are individually housed during transport between the ground and the ISS (Fig. 1b). Supplemental Table l lists the specifications of the new hardware. These two new hardware units allowed us to compare the effects of 1 *g* and μ*g* on mice on the ISS. Creating artificial *g* by centrifugal force makes it possible to have the 1 *g* control in space, where all other conditions are equalised (Fig. 1c,d). Earth’s gravity (1 *g*), the Moon’s gravity (0.16 *g*) and Mars’ gravity (0.38 *g*) can all be mimicked by the rotating the CBEF centrifuge at 77, 31 and 48 rpm, respectively.

Prior to the space experiment, we conducted various ground-based biocompatibility tests of the new hardware, as well as the Coriolis Effect, during short radius-centrifugation onboard and steady state acceleration during the launch and return phases^15–17^. The success of these ground-based biocompatibility tests convinced us to conduct space experiments with our new hardware.

In this report, we present an overview of this space mouse project by the Japan Aerospace Exploration Agency (JAXA) and describe the development of the HCU and TCU. We also present the results of the first mission with onboard 1 *g* control. The JAXA rodent experimental platform ‘MARS’—Multiple Artificial-gravity Research System—presents a novel opportunity to elucidate the specific effects of μ*g* on mice and also enhances our ability to conduct partial *g* experiments in space for future human space exploration.

## Results and Discussion

### Biocompatibility verification tests before the flight experiment

The development of flight hardware for use in space generally includes a step-by-step verification of compatibility with the specific requirements of the space environment by using test models, bread board models, engineering models and qualification models. Therefore, to minimize total cost and time for the development of flight hardware, we verified compatibility by using bread board models and proto-flight models combined with qualification models and flight models. In addition, we separately verified biocompatibility between mice and hardware using bread board models and ground models, which were an identical design to the flight hardware. The proto-flight model was treated as the flight hardware after passing all required qualification tests.

Using the bread board or ground models of the HCU and TCU, four independent long-term biocompatibility verification tests were conducted before the flight experiment (Supplemental Fig. 1). Changes in food consumption were similar between mice in the control cage and the HCU, whereas water intake was 1.4–2.1 times higher in the HCU than in the control cage because of water leakage in some HCU cages caused by a nozzle malfunction during the experiment. The body weights of the TCU/HCU mice were slightly lower than those of the control mice (ranging from 0.7 to 5.0%).

The effects of vibration and shock on C57BL/6 J male mice during the launch and return phases were also evaluated using the TCU prototype. Mouse behavior was monitored during vibration (random vibration, 600 s) or shock (50 *g*, 100–2,000 Hz, seven times), and no observable injuries were detected (Supplemental Video 1; vibration (left) and shock (right). These results suggest that mice can be transported into and out of space without any serious issues.

### The first mission

**Figure 2:**
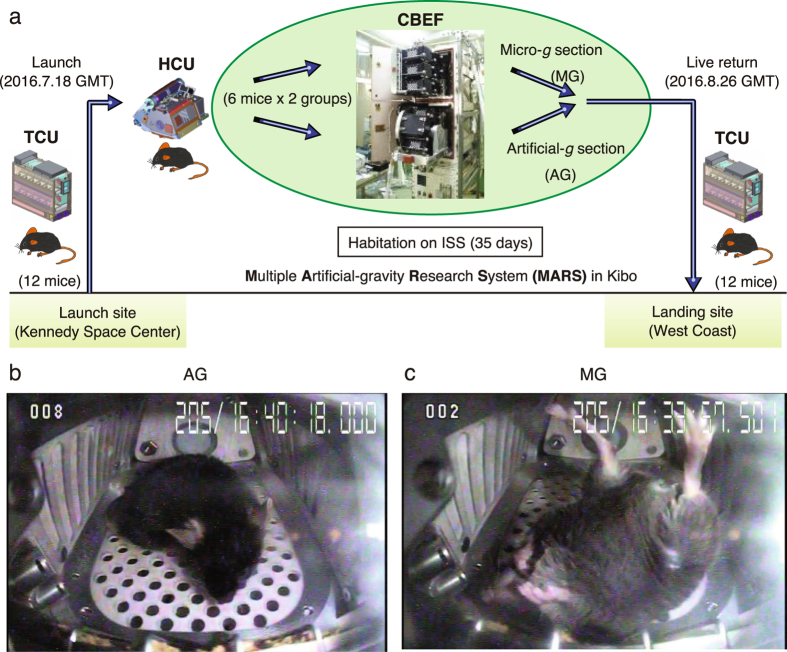
Overview of the first JAXA Mouse Project. (**a**) Twelve male mice (C57BL/6 J) were launched by SpX9 from the Kennedy Space Center on 18 July 2016. On the ISS, the mice were divided into two groups (six mice in MG and six mice in AG). After 35 days of habitation on the ISS, the mice in the TCU were placed into the Dragon capsule and then splashed down in the Pacific Ocean near the West Coast on 26 August 2016. The mice were transported to the laboratory for behavioral observation and dissection two days later.Representative images of onboard habitation for an AG mouse (**b**) and a MG mouse (**c**). Supplemental Videos 2 and 3 show all of the 12 mice onboard.

Figure 2a shows an overview of the first mission. Twelve C57BL/6 J male mice in the TCU were launched aboard SpaceX Falcon 9 rocket (SpX9) on 18 June 2016 from the NASA Kennedy Space Center (KSC) in Florida and then transported to the ISS. The Dragon space vehicle from SpX9 berthed to the ISS on June 20 and mice were transferred to the HCU by the crew. On the ISS, the mice were divided into two groups of six mice each. One group (artificial 1 *g* mice; AG) was kept in an artificial 1 *g* environment (1 *g* on the bottom floor of the HCU, at a centrifugation 77 rpm); the other group (μ*g* mice; MG) was kept under μ*g*. Mice were housed for 34.6 days in the HCU under µ*g* conditions and for 34.1 days and under 1 *g* during the approximate 37-day berthing period. All mice were returned to the Earth in specific pathogen-free conditions (Supplemental Table 2). The ground control experiment replicating the housing conditions of the flight experiment was conducted at the JAXA Tsukuba Space Center in Japan.

### Observation of mouse activity and waste collection onboard

During the onboard habitation, the health condition of each mouse was monitored daily by veterinarians on the ground via downlinked videos. Figure 2b and c show representative images of onboard habitation in the HCU. All 12 mice onboard are shown in Supplemental Videos 2 and 3. The health of each mouse was determined based on the condition of eyes, ears, teeth, fur and tail as observed in videos.

MG mice used their tails to maintain posture while resting. This was quite different from AG mice (Supplemental Video 4). Under the 1 *g* condition, mouse feces easily fell into holes in the HCU floor, thus keeping the HCU clean. Although the gravity-dependent waste collection system does not work under μ*g* conditions, feces were removed from the cage by airflow (Supplemental Video 5, left). Under μ*g* conditions, mice produce waterdrop-like urine, which was absorbed by paper sheets on the bottom/walls of the HCU by airflow (Supplemental Video 5, right). The airflow-dependent cage cleaning maintenance system was very effectively during onboard habitation.

### Environmental parameters in the flight mission

During the flight mission, a temperature/humidity data logger was attached to the TCU during the launch, onboard and return phases and inside the CBEF during the onboard phase. Logger data were processed after the mission. Supplemental Table 3 summarises the data. Average temperatures during the launch, onboard and return phases were 25.8 °C, 23.0 °C and 26.1 °C, respectively. Temperatures higher than 26 °C were observed during the launch and return phases, and lasted 14 and 24.3 h, respectively. The average relative humidity during the launch, onboard and return phases were 36.1%, 46.1% and 41.7%, respectively. The concentrations of carbon dioxide and ammonia were monitored by a sensor inside the CBEF. Concentrations were maintained at low levels during the mission. Supplemental Figs 2a,b and 3a,b show a detailed time course of changes in environmental parameters. The results indicate that mice were housed in a properly controlled environment.

### Level of radiation during the space experiment

As exposure to radiation in space is significantly higher than on the ground, total radiation during this 37-day flight was measured using a ‘Bio Passive Dosimeter for Life Science Experiments in Space’ (Bio PADLES). The absorbed dose rate was 0.23 ± 0.02 mGy/d and the dose equivalent rate was 0.43 ± 0.03 mSv/d during the space experiment. Although the high dose of radiation affects the health of mice, it did not cause an acute response.

### Habitation data from the flight experiment (AG/MG)

**Figure 3:**
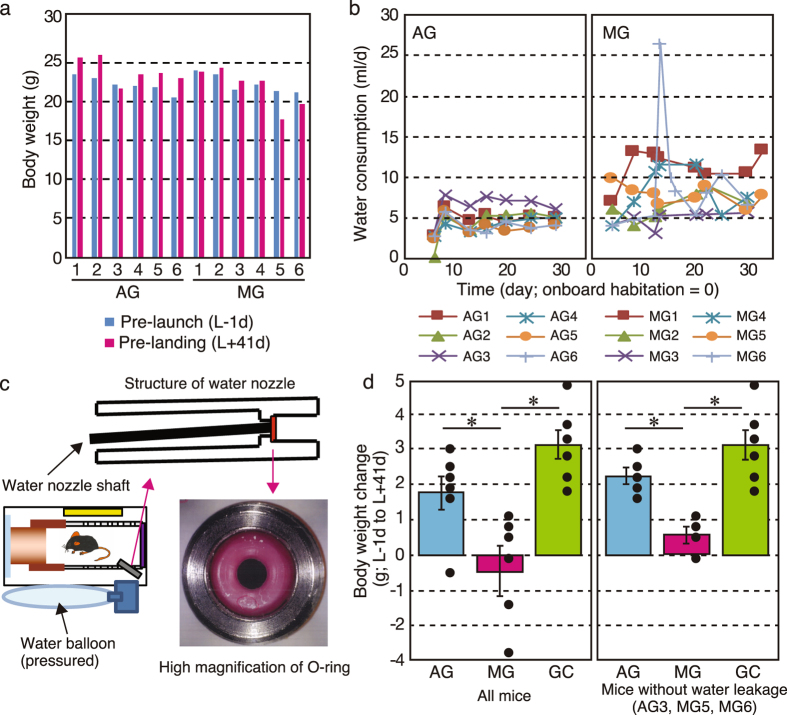
Habitation data on flight mice and the hardware malfunction during the mission.Summarised data on body weight change (**a**) and water intake (**b**). The MG mice either gained some weight or maintained their preflight weight, whereas AG mice gained weight. Because of water leakage in the MG cages during onboard habitation, water consumption was higher in MG than in AG. (**c**) Water leakage in the TCU during the return phase. The cylindrical TCU cage and structure of the water nozzle are shown. An O-ring functions as a seal for pressured water in the balloon. When a mouse accesses the water nozzle shaft for drinking, the nozzle is slightly tilted and the pressurised water leaks out. Corrosion particles (circled with the yellow dotted line) were detected on the seal surface of the O-ring. This may have caused unexpected water leakage without access by the mouse. Corrosion particles were observed in the O-ring of AG3, MG5 and MG6. (**d**) The recalculated body weight between pre-launch (L-1d) and post landing (L + 41d). The body weight data was recalculated for all mice excluding AG3, MG5 and MG6. Data was expressed as mean ± SE. *P < 0.05.

There was no electronic balance on the ISS, thus we could not measure body weight and food/water consumption. The MG mice either gained weight or maintained their preflight weight, whereas AG mice in space gained weight (Fig. 3a). However, the body weights of AG3, MG5 and MG6 decreased compared with their preflight values. Food bar access was monitored by video (Supplemental Fig. 4), but no unique differences were observed between AG3, MG5 and MG6 mice and the others. Actual food consumption could not be analyzed during the flight experiment because of limitations in detecting the mass of food retrieved from the food bar.

Our results showed higher water consumption and lower body mass gain in mice in μ*g* than mice in artificial 1 *g*. This was different from previous flight experiments using rats and the NASA AEM, which showed no changes in body weight, caloric intake and water intake between flight and ground control animals^18^. We currently have no explanation for these differences, but it may be the result of physiological differences between rats and mice. Laboratory rats are much less active in cages than mice. Therefore, space flight might have little effect on the physical activities of rats. In future flight experiments, long-term video observation of mouse behavior in μ*g* versus artificial 1 *g* in our system may reveal the reason.

### Hardware malfunctions and improvements for the next mission

Water intake during onboard habitation was estimated by the crew using a graduated water syringe (Fig. 3b). Sample number ‘6’ for the MG mouse on Day 15 (MG6/Day 15) shows a transient increase in water consumptions. During onboard habitation, water leakage was observed in the HCU four times only under the μ*g* condition (MG5/Day 12, MG6/Day 15, MG4/Day 22 and MG5/Day 25). This was because of mice pulling paper sheets off the walls, and fragments became attached to the water nozzle and triggered leaks. Although the airflow generated by ventilation fans cleaned out mouse waste, including feces, some pieces of the paper sheets were unable to pass through the holes in the HCU. After water leakage was observed, a crew member replaced the HCU with a new one. Based on these observations, we concluded that water leakage was not likely to be a common issue and, therefore, was probably not the cause of decreased body weight observed in AG3, MG5 and MG6 mice.

The other experimentally critical hardware malfunction was water leakage from the TCU during the return phase. After we retrieved the mice on the ground, water balloons in cages in the TCU accommodating MG5, MG6 and AG3 mice were almost empty. In mice, lack of water causes dramatic body weight loss. Therefore, the decreased body weight in AG3, MG5 and MG6 mice is likely due to this malfunction. Further analysis revealed that leakage was caused by corrosion particles breaking the seal on the water nozzle. The particles were generated by crevice corrosion and electrolytic corrosion because of two dissimilar materials (aluminium and stainless steel) on parts associated with the watering unit (Fig. 3c). We have already changed the material of these parts to prevent similar problems in future missions. The average body weight change between pre-launch and post-landing were recalculated (Fig. 3d). Changes in AG mice were slightly less than in GC mice. This difference may be linked to the effects of shock and vibration during the launch and return phases. Otherwise, all mice were exposed to μ*g* just after rocket launch and before splashdown because there was no centrifuge inside the rocket. This uncontrollable μ*g* phase, which lasted up to 3 days, may cause the differences in body weight between AG and GC mice.

### Analysis of returned mice

**Figure 4:**
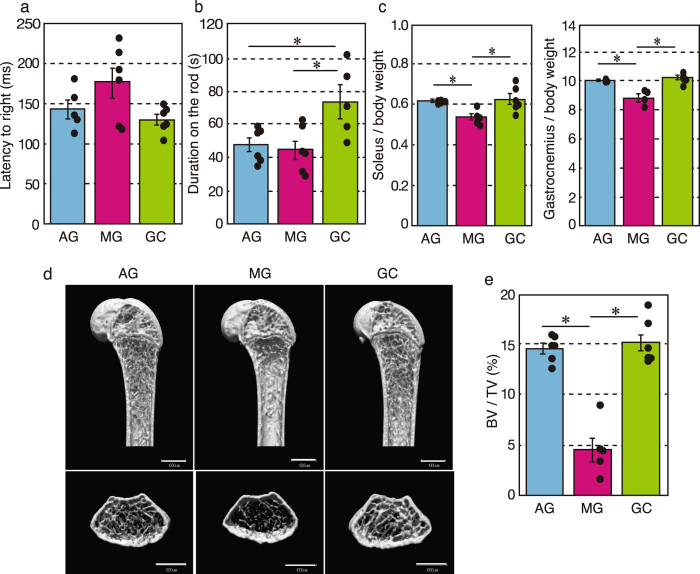
Effects of microgravity on vestibular function, skeletal muscle and bone. (**a**) Mid-air righting reflex test. AG (n = 5), MG (n = 6) and GC (n = 6). (**b**) Rotarod performance test. AG (n = 6), MG (n = 6) and GC (n = 5). *P < 0.05. (**c**) Body-weight-normalized skeletal muscle masses from AG (n = 5), MG (n = 4) and GC (n = 6) mice. *P < 0.05. (**d**) The representative vertical (upper) and horizontal (lower) sectional microCT photos of the proximal region of the femur. Scale bars = 1 mm. (**e**) The calculated cancellous bone volume/tissue volume (BV/TV, %) of the femurs of AG (n = 6), MG (n = 5) and GC (n = 6) mice. *P < 0.05.

To evaluate vestibular function and coordinated movement, a mid-air righting reflex test and rotarod test were conducted after mice returned to the ground. We excluded the mid-air righting data for AG6, because this mouse failed to take an upright posture. Latency to righting in AG and GC mice was similar (143 ± 12 ms and 130 ± 7 ms, respectively), but tended to be delayed in MG mice (176 ± 19 ms), which suggests that the sense self-orientation in space might be impaired in MG mice (Fig. 4a). However, the differences were not statistically significant (F^[2, 14]^ = 3.1, P = 0.0719). Figure 4b shows how long mice remained on the rotating rod. Data from GC5 were excluded, because this mouse did not walk on the rod. AG and MG mice had significantly shorter times on the rotarod than GC mice (F^[2, 14]^ = 5.9, P = 0.0139), which suggests impaired motor coordination in AG and MG mice.

Further phenotypic analysis showed a statistically significant decrease of approximately 90% in the weight of the soleus and gastrocnemius muscles (i.e. antigravity muscles) (F^[2, 14]^ = 5.8, P = 0.017 and F^[2, 12]^ = 24.4, P = 5.97E-05, respectively) compared with AG and GC mice (Fig. 4c). In addition, micro-computed tomography analysis revealed that artificial *g* loading significantly suppressed bone loss induced by μ*g* conditions (F^[2, 14]^ = 42.07, P = 1.2E-06; Fig. 4d,e).

The most significant finding of this study is that the addition of artificial *g* prevents space-induced disuse atrophy. Astronauts on the ISS exercise daily for 2–3 h to maintain their skeletal muscles and bones. However, aside from muscles and bones, we know little about whether exercise is sufficient to maintain tissues and major organs of the body. We are in a new stage of studying how gravity controls and maintains the functions of bones, muscles and other organs. The new JAXA rodent experimental platform provides the opportunity to investigate the specific impacts of μ*g* on mice and to conduct investigations on the effects of partial *g* in space for future human space exploration.

## Methods

### New hardware for the project

#### Mouse HCU

The HCU is an onboard habitation cage that accommodates one mouse per cage. The HCU is equipped with a food bar, watering system (two redundant water nozzles and a water balloon acting as power-free pressure source), an odour filter, two fans (for redundancy) for air ventilation, waste collecting equipment, an LED/IR video camera with a wiper inside the cage to keep the observation window clean (Fig. 1a and Supplemental Video 6). Paper sheets were mounted on the cage wall to quickly eliminate liquid from the cage, such as urine. A photocatalytic thermal spray was applied to the sheets for deodorising and antibacterial effects. Air ventilation inside the cage was maintained by airflow (<0.2 m/s) generated by fans on the HCU. Differences in the volume of air ventilation and airflow rate between HCU in artificial and microgravity conditions is negligible because the fans regulating ventilation in both gravity conditions are maintained at the same speed. The day/night cycle uses 12 h intervals. Nominally, the cages must be replenished once every 2 weeks. Feed and water resupply are required once a week. Logs of temperature, humidity, carbon dioxide and ammonia can be monitored. Supplemental Table 1 lists the detailed specifications of the HCU. For the first mission, 24 HCU were launched to house 12 mice; the remaining 12 HCU were spares in case of problems.

#### TCU

The TCU was used to transport mice aboard the SpaceX Dragon space vehicle in the launch and return phases, and it was placed in a powered locker sized for ISS single cargo transfer. The TCU contains 12 cylindrical cages for housing mice individually. The TCU is equipped with a cylindrical food bar, watering system (two redundant water nozzles and a water balloon), an odour philtre, two fans (for redundancy), waste collecting equipment and LEDs with lights for day and night cycles. Paper sheets mounted in the waste collection area were treated with photocatalytic thermal spray for deodorising and antibacterial effects, similar to the HCU. A temperature/humidity logger was attached on the TCU air inlet to monitor the environment during subsequent operations. Supplies of food and water were sufficient for up to 10 days. Supplemental Table 1 lists the detailed specifications of the TCU.

### Animals

Five-week-old C57BL/6 J male mice (Stock #000664) were purchased from the Jackson Laboratories (USA) for the space experiment and from the Charles River Laboratories (Japan) for ground experiments. All experiments were approved by the Institutional Animal Care and Use Committee of University of Tsukuba (No. 16–048), JAXA (Protocol Number: 016-014B), Explora Biolabs (Study Number: EB15-010A) and NASA (Protocol Number: NAS-15-004-Y1), and experiments were conducted according to the guidelines and applicable laws in Japan and the United States of America. Mice were maintained under specific pathogen-free conditions throughout the space experiment (Supplemental Table 2).

### Biocompatibility verification tests using the prototype or ground models of the HCU and TCU

To simulate the flight experiment, C57BL/6 J male mice (8 weeks old) were sequentially housed in the TCU for 10 days (simulated launch phase), in the HCU (simulated onboard phase) for 30–45 days and then returned to the TCU (simulated return phase) for 5 days. Food/water consumption and body weight changes were measured. The mice were fed CRF-1 (Oriental Yeast Co., Ltd., Tokyo, Japan). The drinking water was autoclaved tap water containing iodine (0.2 mg/l). The bedding material consisted of paper chips (ALPHA-DRI; Shepherd Specialty Papers Inc., Watertown, MA, USA) in the control cages (Inocage; Oriental Giken, Tokyo, Japan) but was not used in the TCU and HCU. Feeder cartridges and water bottles were replaced once a week, but HCU were not replenished. After housing, the health of the mice was checked and their body weights were measured.

### Vibration and shock tests

The effects of vibration and shock on C57BL/6 J male mice (8 weeks old) during the launch phase were evaluated using the TCU prototype. Random vibrations (600 s) or shock (50 *g*, 100–2,000 Hz, 7 times) were applied to mice (n = 3) using the 18-tonne vibration test facility at JAXA.

### Flight schedule

The SpX9 was launched on 18 July 2016 (EDT) from the KSC. After arriving at the ISS, mice were relocated from the TCU to the HCU by the crew, where the animals were housed for 35 days. Before mice were returned to the ground, centrifugation was halted and they were transferred to the SpX9 Dragon capsule. The capsule splashed down in the Pacific Ocean offshore from California on 26 August 2016 (GMT). Following splashdown, the TCU containing mice was unloaded from the SpX9 Dragon capsule and transported to Long Beach Airport on 28 August 2016 (GMT). The TCU was handed over to JAXA at the airport and then transported to a laboratory in San Diego (Explora Biolabs) by an environmentally controlled van. Returned mice were euthanized and dissected at the laboratory to collect tissues. All time points in this paper are expressed relative to rocket-launch (L), except for the daily results of onboard habitation (Fig. 3b).

### Pre-launch acclimation activities and animal selection

Three weeks prior to launch (L-21d), 100 mice (5 weeks old) were delivered from Jackson Lab to KSC (Supplemental Fig. 5a). Then the 100 mice were acclimatized to the environment for three weeks in individual housing cages (Small Mouse Isolator 10027, Lab Products Inc., USA) in an air-conditioned room (temperature: 23 ± 3 °C; humidity: 40–65%) with a 12:12-h light–dark cycle at the SSPF Science Annex at KSC. Three acclimation phases were set: Phase I, body weight recovery phase (L-21d to L-14d); Phase II, water nozzle acclimation (L-14d to L-7d) and Phase III, flight food acclimation (L-7d to launch). After approximately three weeks of acclimation, the mice were ready for transport and reached the age of 8 weeks. Briefly, at Phase I, mice were fed CRF-1 and given water *ad libitum* using ball-type water nozzles. The drinking water was autoclaved tap water. The bedding material consisted of paper chips (ALPHA-DRI). At Phase II, water nozzles are changed to flight nozzles. Finally, at Phase III, the food was changed to flight food (Supplemental Fig. 5a). Fecal collection and body swabs were obtained at L-13d, and these samples were sent to Charles River Laboratories in the USA for PCR-based SPF testing.

During the acclimation phase, body weight and food/water consumption were measured and recorded. Four days prior to launch (L-4d), we divided 78 mice into six subgroups of 13 mice (12 mice for flight and one backup mouse for contingencies such as ear punching failure). The selection of flight candidate mice was based on body weight, food consumption and water intake. We also checked the health of each flight candidate mouse by evaluating the condition of their eyes, ears, teeth, fur and tail. The six subgroups were necessary to support possible launch attempts in case of unexpected postponements. Supplemental Video 7 shows how we evaluated the health of flight mice. A SONY HDR-GWP88 Handycam with 1440 × 1080 resolution (frame width × height) at a rate of 29 frames/s or a CASIO EX-ZR850 Digital Camera with 640 × 480 resolution (frame width × height) at a rate of 29 frames/s were used for shooting videos. All video images were processed by ffmpeg (https://ffmpeg.org/) and/or AviUtl (http://spring-fragrance.mints.ne.jp/aviutl/) and stored as mpeg files.

### Onboard operations by ISS crew

One day prior to launch (L-1d), 12 mice were loaded into the TCU and made ready for launch. Mice were transported to the ISS by SpX9. After the Dragon vehicle of SpX9 berthed to the ISS, mice were transferred to HCU by a crew member. Mouse-keeping tasks included exchanging food cartridges, supplying water, collecting waste and replacing odour filters (Supplemental Fig. 5b). After onboard mouse rearing, mice were transferred from the HCU to the TCU for the return to Earth. All crew tasks onboard the ISS took about 70 h.

### Downlinked video images from the ISS

The camera attached to the HCU was a 1/3-inch interlace CCD image sensor (NTSC) with effective pixels of 768 (H) × 494 (W). Video data from the camera was transmitted to the ground at a transfer rate of 6 Mbps after analog-to-digital conversion. More than 10 min/d of video footage from each cage was downlinked from the ISS for observations on health by ground veterinarians.

### Return phase and animal dissection

After unberthing from the ISS, the Dragon vehicle splashed down in the Pacific Ocean off the coast of California. After a ship picked up the cargo, the returned TCU was transported to a port in Long Beach (Supplemental Fig. 5c). JAXA later received the TCU from NASA and transported it to Explora Biolabs in San Diego for analysis. The health and body weights of mice were checked. Checking on the health of mice, using the same process as for the launch health check, can be seen in Supplemental Video 8. Next, the mid-air righting reflex test was conducted. Each mouse was oriented in spine position (back to the ground) and dropped twice from a height of about 40 cm onto a padded surface. The average time for righting was analyzed using high speed video (DSC-RX100M5, SONY, Tokyo, Japan). The rotarod performance test was conducted after the righting reflex test. Rotation speed was increased from 2 to 40 rpm over 2 min (47600, Bioresearch Center, Nagoya, Japan), and we measured how long mice remained on the rotating rod. This test was performed twice for each mouse. Isoflurane-anesthetised mice were euthanized by exsanguination and dissected for the collection of tissue samples.

### Logging data for environmental parameters

#### Temperature and humidity

Temperature and humidity were recorded by a data logger (DS1923; KN Laboratories, Inc., Osaka, Japan). The logger was attached to the TCU during launch and return phases and set inside the CBEF during the onboard phase.

#### Gas concentrations of carbon dioxide and ammonia onboard

Gas concentrations were monitored every minute by sensors inside the CBEF. The S-300MR (ELT SENSOR Corp.) was used to detect carbon dioxide and the NE-NH3 (Nemoto Sensor Engineering CO., Ltd.) was used to detect ammonia. The resolution was approximately 10 ppm (accuracy: ± 0.3% F.S.) and 0.03 ppm (accuracy: ± 1.6% F.S.), respectively.

#### Radiation

During this 37-day flight, we measured exposure to two kinds of energy by physical dosimetry using Bio PADLES containing four plates of CR-39 plastic nuclear track detectors and seven thermoluminescent dosimeter elements^19^. Two Bio PADLES packages were prepared: one was attached on the TCU during the launch and return phases and moved to the CBEF during the onboard habitation phase; the other was kept on the ground throughout the entire mission. Results were expressed as the absorbed dose rate (mGy/d) and dose equivalent rate (mSv/d) calculated with quality factors as a function of LET_∞_, H_2_O as recommended in the ICRP60.

### Ground control experiment

A ground control experiment that simulates the space experiment was conducted at JAXA Tsukuba in Japan from August 31 to 3 November 2016. Six mice (Stock #000664, 5 weeks old, Charles River Japan) were individually housed in the TCU for 5 days, in the HCU for 35 days and in the TCU for 4 days. Both the TCU and HCU were placed in an air-conditioned room (temperature: 24 ± 2 °C; humidity: 40–65%) with a 12:12-h light–dark cycle. Fan-generated airflow (0.2 m/s) inside the HCU maintained the same conditions as in the flight experiment. The mice were fed CRF-1. The drinking water was autoclaved tap water containing iodine (0.2 mg/l). The bedding material consisted of paper chips (ALPHA-DRI) in the control cages, but was not used in the HCU. Feeder cartridges and water bottles were replaced once a week, and cages in the HCU were not replenished. The health of the mice were checked and their body weights measured. Next, the mid-air righting reflex test and rotarod performance test were conducted. All mice were euthanized by inhaling lethal doses of isoflurane and then dissected to collect tissue samples.

### Micro-computed tomography analysis

The right femurs of mice were fixed in 70% ethanol and proximal regions were analyzed. MicroCT scanning was performed using a ScanXmate-A100S Scanner (Comscantechno, Yokohama, Japan). Three-dimensional micro-structural image data was reconstructed and bone volume/tissue volume (BV/TV, %) was calculated using TRI/3D-BON software (RATOC System Engineering, Tokyo, Japan) in accordance with the guidelines^20^.

### Statistical analysis

All data are presented as means ± SEM. All data were analyzed using one-way analysis of variance by group (AG vs. MG, AG vs. GC and MG vs. GC).

## Electronic supplementary material

Supplemental Information

Supplemental Video 1

Supplemental Video 2

Supplemental Video 3

Supplemental Video 4

Supplemental Video 5

Supplemental Video 6

Supplemental Video 7

Supplemental Video 8
