# Supplementary material for: Development of new experimental platform ‘MARS’—Multiple Artificial-gravity Research System—to elucidate the impacts of micro/partial gravity on mice
Source: Sci Rep. 2017 Sep 7;7:10837. doi: 10.1038/s41598-017-10998-4 (PMC5589811; doi:10.1038/s41598-017-10998-4)
Supplement: Supplementary file 1 — Supplemental Information [file 41598_2017_10998_MOESM1_ESM.pdf]

Supplemental Information

- Supplemental Figures (1–5)
- Supplemental Videos (#1–#8)
- Supplemental Tables (1–3)

Title

Development of new experimental platform 'MARS'—Multiple Artificial-gravity Research System—to elucidate the impacts of micro/partial gravity on mice

Author list and affiliations

Dai Shiba,[1, 2]* Hiroyasu Mizuno,[1, 2] Akane Yumoto,[1, 2] Michihiko Shimomura,[1, 2] Hiroe Kobayashi,[1, 2] Hironobu Morita,[1, 3] Miki Shimbo,[1, 4, 5] Michito Hamada,[1, 4, 5] Takashi Kudo,[1, 4, 5] Masahiro Shinohara,[1, 7, 8] Hiroshi Asahara,[1, 7] Masaki Shirakawa,[1, 2]* and Satoru Takahashi[1, 4, 5, 6]

1) Mouse Epigenetics Project, ISS/Kibo experiment, Japan Aerospace Exploration Agency (JAXA)
2) JEM Utilization Center, Human Spaceflight Technology Directorate, JAXA
3) Department of Physiology, Gifu University Graduate School of Medicine
4) Department of Anatomy and Embryology, Faculty of Medicine, University of Tsukuba
5) Laboratory Animal Resource Center, Faculty of Medicine, University of Tsukuba
6) Transborder Medical Research Center, Faculty of Medicine, University of Tsukuba
7) Department of Systems BioMedicine, Graduate School of Medical and Dental Sciences, Tokyo Medical and Dental University
8) Japan Science and Technology Agency (JST), Precursory Research for Embryonic Science and Technology (PRESTO)

*Corresponding author:

Dai Shiba, Ph.D., JEM Utilization Center, Human Spaceflight Technology Directorate, JAXA, Tsukuba, Ibaraki 305-8505, Japan.

Tel: +81-50-3362-2734; Fax: +81-29-868-3950; E-mail: shiba.dai@jaxa.jp

Masaki Shirakawa, Ph.D., JEM Utilization Center, Human Spaceflight Technology Directorate, JAXA, Tsukuba, Ibaraki 305-8505, Japan.

Tel: +81-50-3362-6307; Fax: +81-29-868-3950; E-mail: shirakawa.masaki@jaxa.jp

<u>Supplemental Material Information</u>

Supplemental Figures

Experiment	Launch phase simulation (TCU)	Onboard phase simulation (HCU)	Return phase simulation (TCU)	Summary: Food consumption (HCU phase)	Summary: Water intake (HCU phase)	Summary: Body weight
#1 (n=6)	Control 10 days	Control 30 days	Control 5 days	Cont 3.58g (AVE) Test 3.47g (AVE)	Cont 3.89g (AVE) Test 5.28g (AVE) 1.4 times higher	Cont 28.2g±2.2 Test 26.8g±1.5 (Δ-5.0%)
	prototype 10 days	BBM 30 days	prototype 5 days			
#2 (n=6)	Control 10 days	Control 45 days	Control 5 days	Cont 3.33g (AVE) Test 3.21g (AVE)	Cont 3.80g (AVE) Test 6.65g (AVE) 1.8 times higher	Cont 28.0g±1.1 Test 27.4g ±1.0 (Δ-2.2%)
	prototype 10 days	GM 45 days	prototype 5 days			
#3 (n=6)	Control 10 days	Control 29 days	Control 5 days	Cont 3.35g (AVE) Test 3.27g (AVE)	Cont 3.79g (AVE) Test 7.78g (AVE) 2.1 times higher	Cont 27.5g±0.7 Test 27.3g ±0.6 (Δ-0.7%)
	GM 10 days	GM 29 days	GM 5 days			
#4 (n=6)	Control 10 days	Control 45 days	Control 5 days	Cont 3.27g (AVE) Test 3.24g (AVE)	Cont 4.16g (AVE) Test 7.40g (AVE) 1.8 times higher	Cont 28.1g±2.0 Test 27.1g ±0.5 (Δ-3.6%)
	GM 10 days	BBM 45 days	GM 5 days			

Supplemental Figure 1

Summary of biocompatibility verification tests. Four independent long-term tests were conducted. Control, typical control cage; prototype, prototype cage; BBM, bread board model cage; GM, ground model cage.

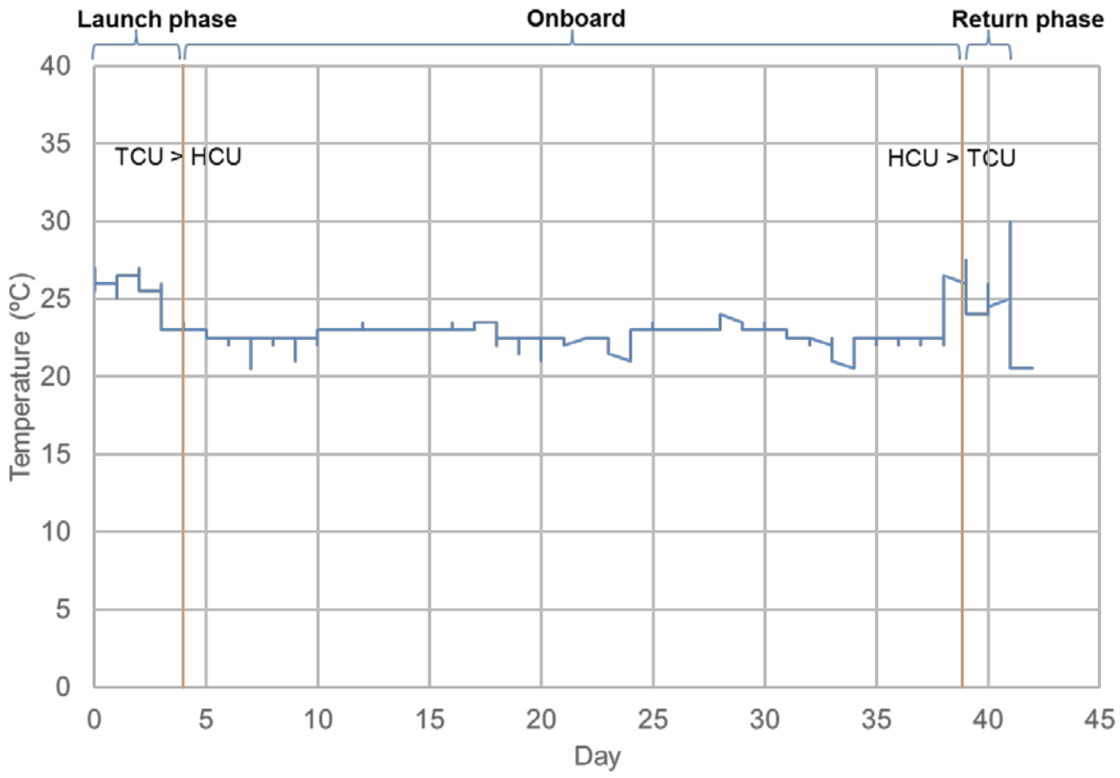

Supplemental Figure 2a

Changes in temperature during the mission.

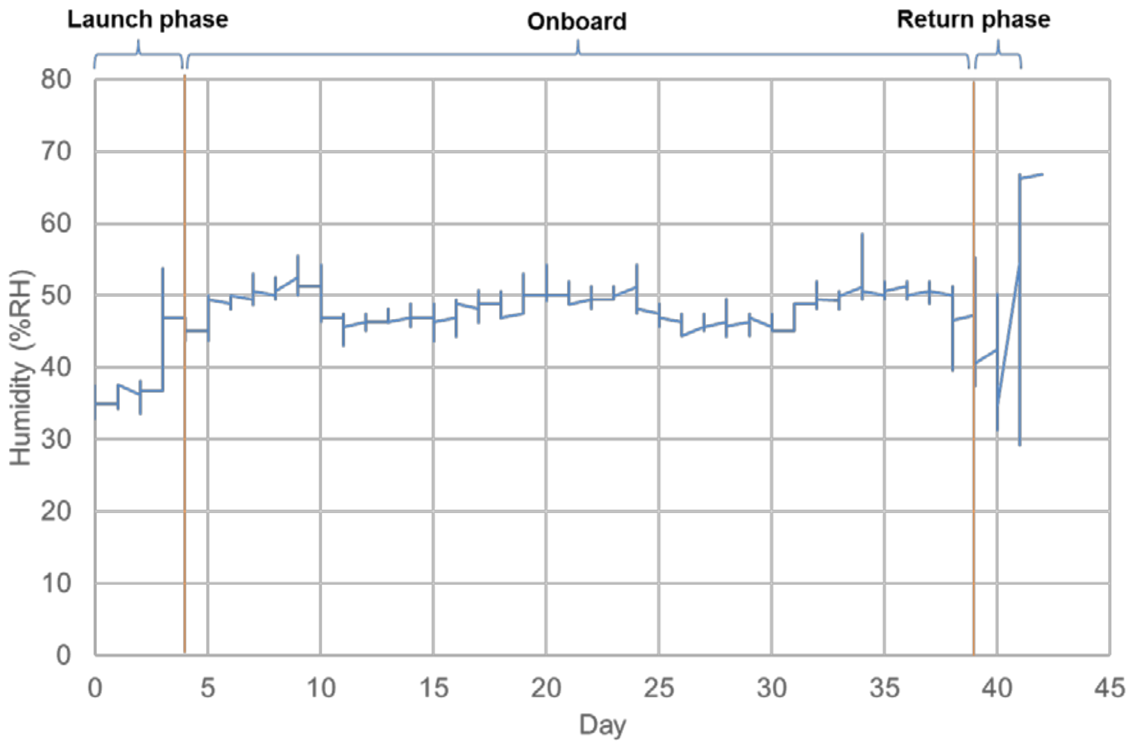

Supplemental Figure 2b

Changes in humidity during the mission.

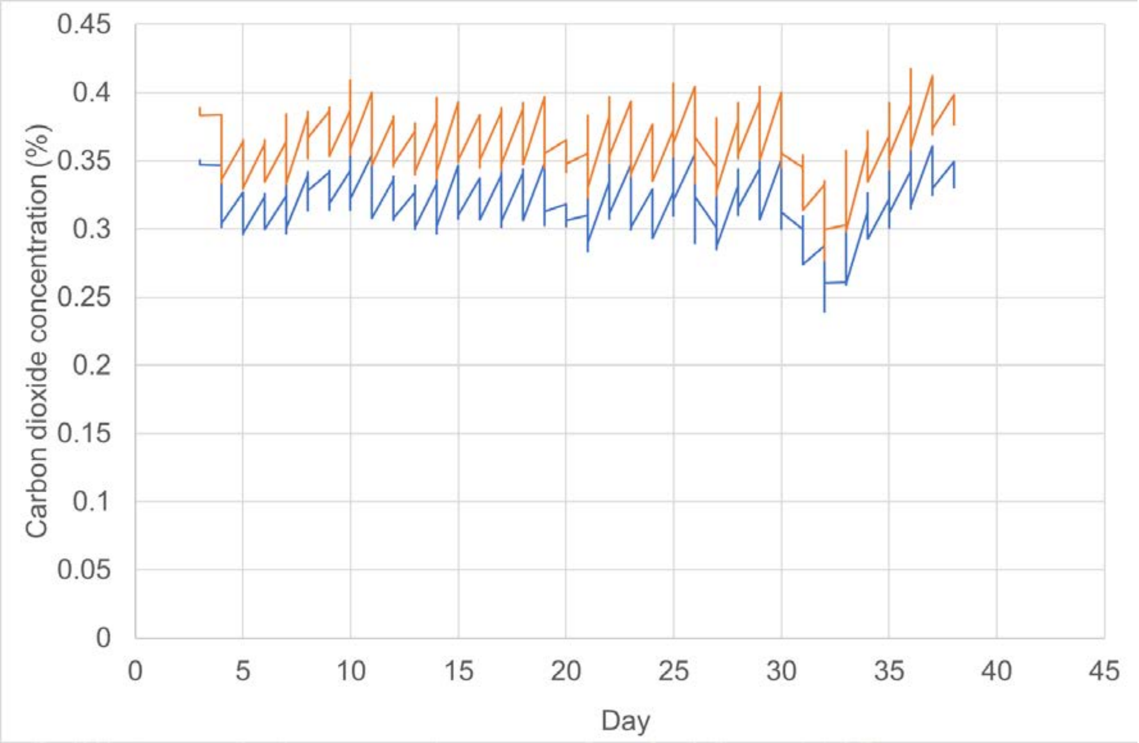

Supplemental Figure 3a

Changes in carbon dioxide concentration during the onboard housing. (Blue line, artificial *g* section; orange line, μ*g* section)

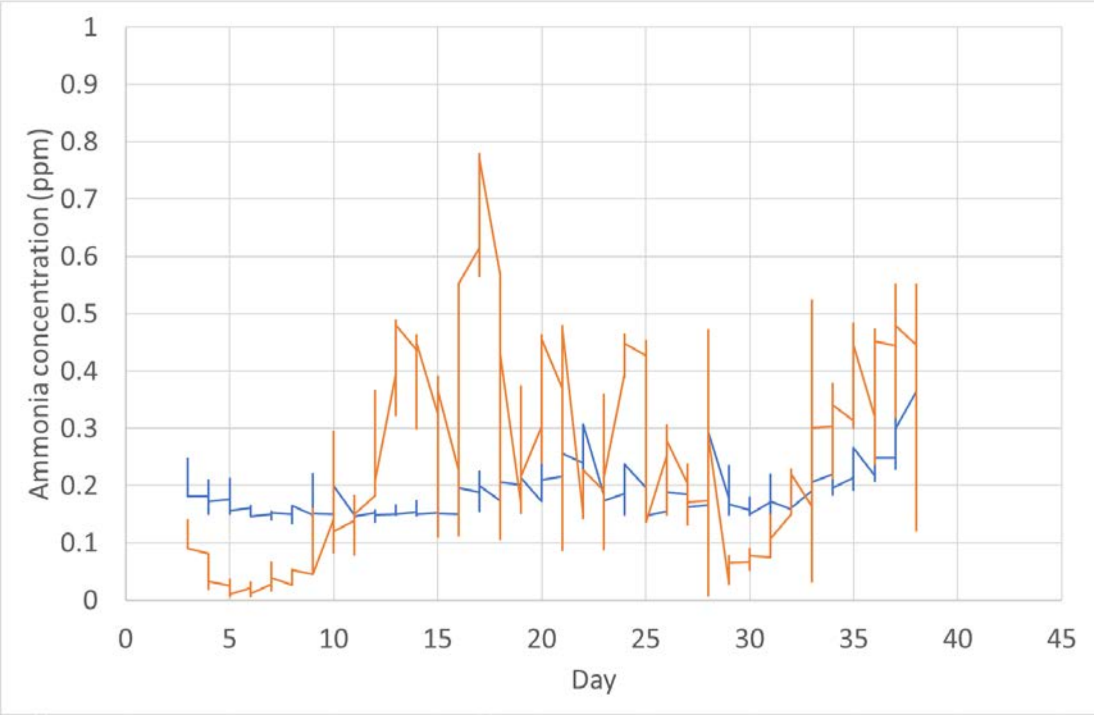

Supplemental Figure 3b

Changes in the concentration of ammonia during onboard housing. (Blue line, artificial *g* section; orange line, μ*g* section)

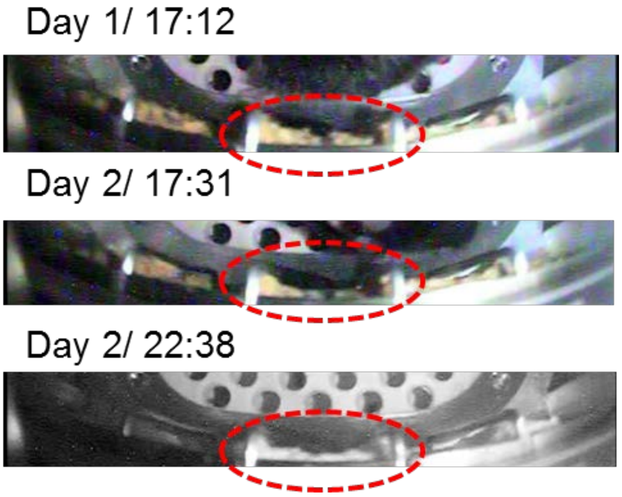

Supplemental Figure 4

Method of checking for daily food bar access during onboard habitation. The dotted red line indicates the area where a mouse could consume food. Changes in the food bar area indicate that the food was accessed.

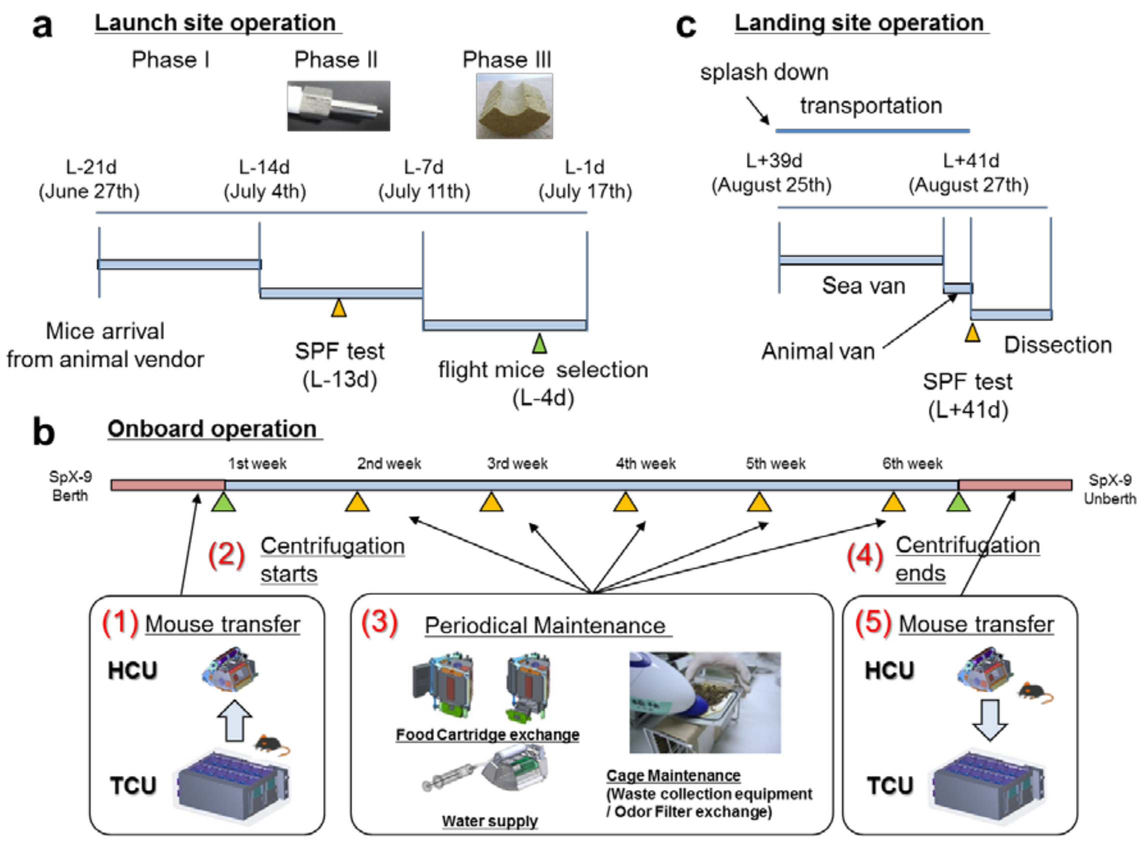

Supplemental Figure 5

Detailed schedule of pre-launch, onboard and post landing events.

(**a**) Schedule for acclimation at the launch site (Kennedy Space Center, Florida). The three acclimation phases: Phase I: body weight recovery phase, Phase II: water nozzle acclimation and Phase III: flight food acclimation. Each phase lasts about 7 days. During acclimation, the SPF test was conducted using faeces and body swabs.

(**b**) After arriving at the ISS, 12 mice were transferred from the TCU to the HCU by crew (1) and centrifugation was started (2). During onboard housing, weekly maintenance was performed by the crew (3). Before mice were returned to the ground, centrifugation was halted and they were transferred to the TCU. Mission duration (SpX9 berthing) was approximately 37 days and the centrifugation period was 34.6 days.

(**c**) After 37 days of habitation on the ISS, mice aboard the SpX9 Dragon spacecraft splashed down in the Pacific Ocean near the West Coast. A ship picked up the SpX9 Dragon capsule containing the TCU, which was then transported to the Long Beach port inside the Sea Van. The TCU was transferred to the laboratory by an animal van for dissection.

Supplemental Videos

Supplemental Video #1

A video of typical mouse behaviour during vibrations (left) and shock (right) (7 s).

Supplemental Video #2

A video of onboard habitation by AG and MG mice at the beginning of the mission (Day 4) (10 s). Panel 1: AG1, AG2 and AG3 (left to right); panel 2: AG4, AG5 and AG6 (left to right); panel 3: MG1, MG2 and MG3 (left to right) and; panel 4: MG4, MG5 and MG6 (left to right). In the video, the numbers 007–012 correspond to AG1–AG6 and the numbers 001–006 correspond to MG1–MG6.

Supplemental Video #3

A video of onboard habitation by AG and MG mice at the beginning of the mission (Days 22 and 23) (10 s). Panels 1: AG1, AG2 and AG3 (left to right); Panel 2: AG4, AG5 and AG6 (left to right); panel 3: MG1, MG2 and MG3 (left to right) and; panel 4: MG4, MG5 and MG6 (left to right). In the video, the numbers 007–012 correspond to AG1–AG6 and 001–006 correspond to MG1–MG6.

Supplemental Video #4

A video of the typical resting posture of an AG mouse (left) and an MG mouse (right) (10 s).

Supplemental Video #5

A video of mouse faeces (left) being removed from the cage and a video of urine (right) being absorbed into paper sheets in the bottom/walls of the cage by airflow in an MG cage (10 s).

Supplemental Video #6

A video of the wiper inside the cage cleaning the observation window in an MG cage (60 s).

Supplemental Video #7

A video of checking the health of AG and MG flight mice on launch minus 4 days (L-4d). Panel 1: AG1, AG2 and AG3 (left to right); panel 2: AG4, AG5 and AG6 (left to right); panel 3: MG1, MG2 and MG3 (left to right); panel 4: MG4, MG5 and MG6 (left to right).

Supplemental Video #8

A video of checking the health of AG and MG flight mice after return (L+41d). Panels 1: AG1, AG2

and AG3 (left to right); panel 2: AG4, AG5 and AG6 (left to right); panel 3: MG1, MG2 and MG3 (left to right); panel 4: MG4, MG5 and MG6 (left to right).

<u>Supplemental Tables</u>

Supplemental Table 1

	HCU	TCU
Size	Floor area: 1.022×10^{-2} m^2<br>Diagonal length: 0.127 m	Diameter: 0.055 m<br>Length 0.154 m
Water	Filtered sterilized water	
Water Tank Maximum Volume	70 ml	140 ml
Water Nozzle	A1400 (Edstrom Industries, Inc.)	
Food type/weight	Block/35 g, Cartridge type	Cylindrical/70 g
Camera	PA-290ADN<br>(Star Micro Co. Ltd.)	N/A
LED	50 lux (12-12 h light dark cycle)	60 lux (12-12 h light dark cycle)
Temperature sensor	CRZ-2005-100 (Hayashi Denko co Ltd.)	A data logger (DS1923; KN Laboratories, Inc.) was attached on the TCU.
FAN	Volume of air ventilation: 8.8l/min<br>Wind velocity: 0.2 m/s in maximum	Volume of air ventilation: 8.8l/min<br>Wind velocity: 0.2 m/s in maximum
WALL	Cover and Liquid absorbing paper<br>Cover) Material: Polycarbonate<br>Liquid absorbing paper) PulSheet Oriental Yeast Co., ltd	Cover and Liquid absorbing paper<br>Cover) Material: Polycarbonate<br>Liquid absorbing paper) PulSheet Oriental Yeast Co., ltd
Odor filter	Activated carbon	Activated carbon

Supplemental Table 2

MICROORGANISM	Pre-launch sample	Onboard sample	Post-landing sample
Zoonotic Viruses			
Lymphocytic Choriomeningitis Virus (LCMV)	Negative (Fecal PCR)	Negative (Fecal PCR)	Negative (Fecal PCR)
Hantavirus	Negative (Fecal PCR)	Negative (Fecal PCR)	Negative (Fecal PCR)
Non-Zoonotic Viruses			
Minute Virus of Mice (MVM)	Negative (Fecal PCR)	Negative (Fecal PCR)	Negative (Fecal PCR)
Mouse Group-A Rotavirus (EDIM)	Negative (Fecal PCR)	Negative (Fecal PCR)	Negative (Fecal PCR)
Mouse Hepatitis Virus (MHV)	Negative (Fecal PCR)	Negative (Fecal PCR)	Negative (Fecal PCR)
Mouse Parvovirus (MPV)	Negative (Fecal PCR)	Negative (Fecal PCR)	Negative (Fecal PCR)
Murine Norovirus (MNV)	Negative (Fecal PCR)	Negative (Fecal PCR)	Negative (Fecal PCR)
Mouse Adenovirus (MAV)	Negative (Fecal PCR)	Negative (Fecal PCR)	Negative (Fecal PCR)
Theiler's Mouse Encephalomyelitis Virus (TMEV)	Negative (Fecal PCR)	Negative (Fecal PCR)	Negative (Fecal PCR)
Zoonotic Bacteria			
Campylobacter spp.	Negative (Fecal PCR)	Negative (Fecal PCR)	Negative (Fecal PCR)
Leptospira spp.	Negative (Fecal PCR)	Negative (Fecal PCR)	Negative (Fecal PCR)
Salmonella spp.	Negative (Fecal PCR)	Negative (Fecal PCR)	Negative (Fecal PCR)

Streptobacillus moniliformis	Negative (Fecal PCR)	Negative (Fecal PCR)	Negative (Fecal PCR)
Non-Zoonotic Bacteria			
Clostridium piliforme (Tyzzer's Disease)	Negative (Fecal PCR)	Negative (Fecal PCR)	Negative (Fecal PCR)
Helicobacter spp.	Negative (Fecal PCR)	Negative (Fecal PCR)	Negative (Fecal PCR)
Pasturella pneumotropica	Negative (Fecal PCR)	Negative (Fecal PCR)	Negative (Fecal PCR)
Zoonotic Ecto Parasites			
Fur mite – *Mobia musculi*	Negative (PCR/ Body swab)	N/A	N/A
Fur mite – *Myocoptes musculinus*	Negative (PCR/ Body swab)	N/A	N/A
Fur mite – *Radfordia spp.*	Negative (PCR/ Body swab)	N/A	N/A
Zoonotic Endo Parasites			
Protozoan – *Giardia spp.*	Negative (Fecal PCR)	Negative (Fecal PCR)	Negative (Fecal PCR)
Protozoan – *Spironucleus spp.*	Negative (Fecal PCR)	Negative (Fecal PCR)	Negative (Fecal PCR)
Non-Zoonotic Endo Parasites			
Pinworm – *Syphacia obvelata*	Negative (Fecal PCR)	Negative (Fecal PCR)	Negative (Fecal PCR)
Pinworm – *Aspiculuris tetráptera*	Negative (Fecal PCR)	Negative (Fecal PCR)	Negative (Fecal PCR)

Supplemental Table 3

Items	Data source	Launch	On-orbit		Return	Remarks
			AG	MG		
Temperature (˚C)	CBEF	N/A	22-24.5 (AVE 23.1)	22-24 (AVE 23.1)	N/A	
	TCU	24-27 (AVE 25.8)	20.5-24 (AVE 22.7)		24-30 (AVE 26.1)	Higher than 26˚C: 14 h for launch, 24.3 h for return
	HCU telemetry	N/A	22.8-23.8 (AVE 23.1)	22.5-24 (AVE 22.7)	N/A	
Humidity (Relative humidity; %)	CBEF	N/A	42-48 (AVE 44.7)	43-52 (AVE 47.4)	N/A	
	TCU	32.9-42.5 (AVE 36.1)	43.1-50.5 (AVE 48.6)		29.2-55.3 (AVE 41.7)	Less than 30%: 100 min for return
Carbon dioxide concentration (%)	HCU telemetry	N/A	0.24-0.37 (AVE 0.32)	0.28-0.42 (AVE 0.36)	N/A	
Ammonia concentration (ppm)	HCU telemetry	N/A	0-0.6 (AVE 0.18)	0-0.88 (AVE 0.25)	N/A	
